# Streptococcus mitis and S. oralis Lack a Requirement for CdsA, the Enzyme Required for Synthesis of Major Membrane Phospholipids in Bacteria

**DOI:** 10.1128/AAC.02552-16

**Published:** 2017-04-24

**Authors:** Hannah M. Adams, Luke R. Joyce, Ziqiang Guan, Ronda L. Akins, Kelli L. Palmer

**Affiliations:** a Department of Biological Sciences, The University of Texas at Dallas, Richardson, Texas, USA; b Department of Biochemistry, Duke University Medical Center, Durham, North Carolina, USA; c Methodist Charlton Medical Center, Dallas, Texas, USA

**Keywords:** daptomycin, Streptococcus, lipidomics

## Abstract

Synthesis and integrity of the cytoplasmic membrane are fundamental to cellular life. Experimental evolution studies have hinted at unique physiology in the Gram-positive bacteria Streptococcus mitis and S. oralis. These organisms commonly cause bacteremia and infectious endocarditis (IE) but are rarely investigated in mechanistic studies of physiology and evolution. Unlike in other Gram-positive pathogens, high-level (MIC ≥ 256 μg/ml) daptomycin resistance rapidly emerges in S. mitis and S. oralis after a single drug exposure. In this study, we found that inactivating mutations in *cdsA* are associated with high-level daptomycin resistance in S. mitis and S. oralis IE isolates. This is surprising given that *cdsA* is an essential gene for life in commonly studied model organisms. CdsA is the enzyme responsible for the synthesis of CDP-diacylglycerol, a key intermediate for the biosynthesis of all major phospholipids in prokaryotes and most anionic phospholipids in eukaryotes. Lipidomic analysis by liquid chromatography-mass spectrometry (LC-MS) showed that daptomycin-resistant strains have an accumulation of phosphatidic acid and completely lack phosphatidylglycerol and cardiolipin, two major anionic phospholipids in wild-type strains, confirming the loss of function of CdsA in the daptomycin-resistant strains. To our knowledge, these daptomycin-resistant streptococci represent the first model organisms whose viability is CdsA independent. The distinct membrane compositions resulting from the inactivation of *cdsA* not only provide novel insights into the mechanisms of daptomycin resistance but also offer unique opportunities to study the physiological functions of major anionic phospholipids in bacteria.

## INTRODUCTION

Streptococcus mitis and S. oralis are human oral colonizers, opportunistic pathogens, and species of the viridans group streptococci (VGS). VGS are associated with ∼23% of Gram-positive bacteremia in immunocompromised patients (1, 2) and ∼17% of infective endocarditis (IE) cases (3). The mitis group VGS are difficult to accurately identify to the species level in clinical settings (4, 5). However, retrospective molecular studies have determined that S. mitis and S. oralis are major causative agents of VGS bacteremia and IE (6–9). Although these organisms are unquestionably significant for human health, very little is known about the physiology and virulence of S. mitis and S. oralis. This is in part due to their close phylogenetic relationship with S. pneumoniae (10–15), to which S. mitis and S. oralis are often compared; they are rarely considered in their own right.

Recent research hints at unique physiology in S. mitis and S. oralis (16–18). Daptomycin (DAP) is a cyclic lipopeptide antibiotic with potent activity against Gram-positive bacterial pathogens, including vancomycin-resistant enterococci (VRE) and methicillin-resistant Staphylococcus aureus (MRSA) (19, 20). Akins et al. reported that high-level DAP resistance (defined as a MIC of ≥256 μg/ml) emerged in S. mitis and S. oralis IE isolates after a single DAP exposure in a simulated endocardial vegetation model (17). High-level DAP resistance after a single drug exposure was also reported by García-de-la-Mària et al. (16) for a collection of mitis group VGS. These results are surprising because DAP resistance in VRE, MRSA, and other model Gram-positive organisms typically emerges in a stepwise fashion by mutation accretion over days or weeks of DAP exposure (21, 22). We infer that DAP resistance in S. mitis and S. oralis proceeds through a novel genetic mechanism compared to those in other Gram-positive bacteria.

In this investigation, we use experimental evolution studies and lipidomics to show that one-step high-level DAP resistance in S. mitis and S. oralis is associated with loss-of-function mutations in *cdsA*, a gene for phospholipid biosynthesis that is essential for Escherichia coli (23), Bacillus subtilis (24), Staphylococcus aureus (25), better-studied streptococci (including S. pneumoniae [26–29]), and an organism with an engineered minimal genome (30). We conclude that S. mitis and S. oralis possess unique physiology, allowing DAP resistance to emerge by a novel mechanism.

## RESULTS

### Emergence of high-level DAP resistance in S. mitis and S. oralis.

The IE clinical isolates S. mitis strain 1643 and S. oralis strains 1647 and 1648 achieved high-level DAP resistance after a single DAP exposure in both simulated endocardial vegetation and bacteremia models (17). We performed serial passage experiments to determine whether DAP resistance would similarly emerge under standard laboratory culture conditions. Parental strains were cultured with DAP (2, 4, 8, and 16 μg/ml). After 24 h of incubation, growth was observed in each inoculated well, regardless of the DAP concentration. Resistant strains obtained from these cultures are referred to as S. mitis 1643-HA04 (derived from parental strain 1643), S. oralis 1647-HA06 (derived from 1647), and S. oralis 1648-HA08 (derived from 1648) (Table 1). We conclude that high-level DAP resistance emerges in S. mitis and S. oralis IE isolates after a single DAP exposure, regardless of the incubation or environmental conditions.

**TABLE 1:** DAP MICs and CdsA details for S. mitis and S. oralis strains used in this study

Strain	Strain description^*a*^	DAP MIC (μg/ml)^*b*^	CdsA change^*c*^
S. mitis 1643	Wild-type IE isolate	0.75	(Q31)
S. mitis 1643-HA04	DAP resistant	>256	*31
S. mitis 1643-HA14	DAP revertant	0.75	W31
			
S. oralis 1647	Wild-type IE isolate	1	(G246)
S. oralis 1647-HA06	DAP resistant	>256	C246
S. oralis 1647-HA16	DAP revertant	1.5	S246
			
S. oralis 1648	Wild-type IE isolate	<0.5	(D249)
S. oralis 1648-HA08	DAP resistant	>256	N249
S. oralis 1648-HA18	DAP revertant	<0.5	D249

a DAP-resistant strains were obtained after wild-type strains were exposed to DAP. DAP revertants were obtained by passage of DAP-resistant strains in drug-free medium.

b MICs were determined by Etest on MHA plates.

c Wild-type amino acids are shown in parentheses. * indicates a stop codon.

DAP resistance confers a modest growth defect on S. mitis and S. oralis. In standard laboratory medium, the resistant strains grew with generation times ≥20% longer than those of parental strains (see Fig. S1 in the supplemental material).

### Mutations in *cdsA* occur in DAP-resistant strains.

**FIG 1:**
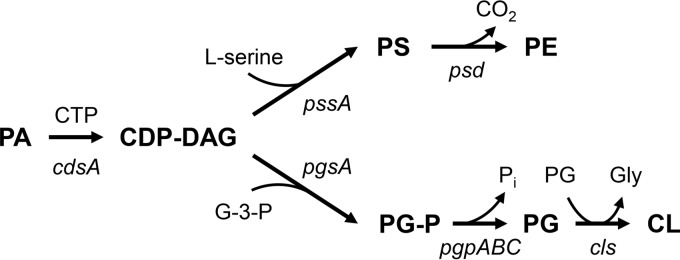
Biosynthetic pathway of the major phospholipids in bacteria. Abbreviations: PA, phosphatidic acid; CDP-DAG, CDP-diacylglycerol; PS, phosphatidylserine; PE, phosphatidylethanolamine; G-3-P, glycerol-3-phosphate; PG-P, phosphatidylglycerol-3-P; PG, phosphatidylglycerol; CL, cardiolipin.

We used genome sequencing to identify mutations in the DAP-resistant strains (Table 2). Nonsynonymous mutations in *cdsA* occurred in all three strain pairs. CdsA catalyzes the conversion of phosphatidic acid (PA) into CDP-diacylglycerol (CDP-DAG), which is the precursor for synthesis of the major membrane phospholipids, including phosphatidylglycerol (PG), cardiolipin (CL), phosphatidylethanolamine (PE), and phosphatidylserine (PS) (Fig. 1).

**TABLE 2:** Mutations occurring in DAP-resistant strains

Strain	Description of gene	Nucleotide variation in gene^*a*^	Amino acid change	Locus^*b*^
S. mitis 1643-HA04	Hypothetical	G531T (98)	S177R	SK608_0120 (100, 100)
	Serine/threonine kinase	A526C (90)	K176Q	smi_1622 (45, 29)
	Ribosomal S5p alanine acyltransferase	G502T (98)	H168N	TZ92_01497 (100, 99)
	**Phosphatidate cytidylyltransferase (CdsA)**^*c*^	**G91A (99)**	**Q31Stop**	**smi_1854 (100, 81)**
S. oralis 1647-HA06	Intergenic region 13	C to T (99)	N/A	9 bp between SOR_1153 and SOR_1154
	Intergenic region 17	A to C (99)	N/A	47 bp upstream of SOR_0277
	**Phosphatidate cytidylyltransferase (CdsA)**	**C736A (97)**	**G246C**	**SOR_1730 (100, 96)**
S. oralis 1648-HA08	ppGpp synthetase	A2075C (96)	V692G	SOR_1513 (100, 99)
	Helicase PriA	T77G (93)	E26A	SOR_1544 (100, 96)
	Ribosomal SSU methyltransferase	C454A (96)	V152F	SOR_1542 (100, 95)
	**Phosphatidate cytidylyltransferase (CdsA)**	**C745T (97)**	**D249N**	**SOR_1730 (100, 96)**

a The frequency of the mutation in the read assembly is shown in parentheses.

b Loci were identified using protein BLAST. Query coverage and percent amino acid identity are shown in parentheses. References used were S. oralis Uo5 (SOR_) (53), S. mitis B6 (smi_) (13), S. mitis SK608 (SK608_) (54), and S. oralis SK141 (TZ92_) (55).

c Bold type indicates that the mutation reverted after serial passage without DAP.

We analyzed the *cdsA* mutations to predict their impacts on CdsA function. S. mitis 1643-HA04 possesses a G91A transition that generates a premature stop codon after the 31st amino acid in the polypeptide chain. We predict that this mutation results in a complete loss of CdsA function in S. mitis 1643-HA04. S. oralis 1648-HA08 possesses a C745T transition that alters the 249th residue from aspartic acid to asparagine. A recently determined crystal structure of CdsA from Thermotoga maritima (TmCdsA) identified residues necessary for TmCdsA function (31). D249 of the streptococcal CdsA aligns to an active-site residue (D246) in TmCdsA which was shown to be required for CdsA activity (31). Finally, S. oralis 1647-HA06 possesses a C736A transversion that converts the 246th residue from a glycine to a cysteine. Analysis of putative secondary structures for the peptide chain using the garnier tool in EMBOSS (32) revealed that G246 in 1647-HA06 occurs in a bend in the peptide (data not shown). Altering the flexible glycine residue to a rigid cysteine residue may prevent the polypeptide chain from folding properly. In summary, *cdsA* mutations in the DAP-resistant strains likely result in CdsA proteins that have loss of function by altering an active site (S. oralis 1648-HA08), preventing proper protein folding (S. oralis 1647-HA06), or by preventing synthesis of the full polypeptide (S. mitis 1643-HA04).

### Lipidomic analysis confirms CdsA loss of function in DAP-resistant S. mitis and S. oralis.

**FIG 2:**
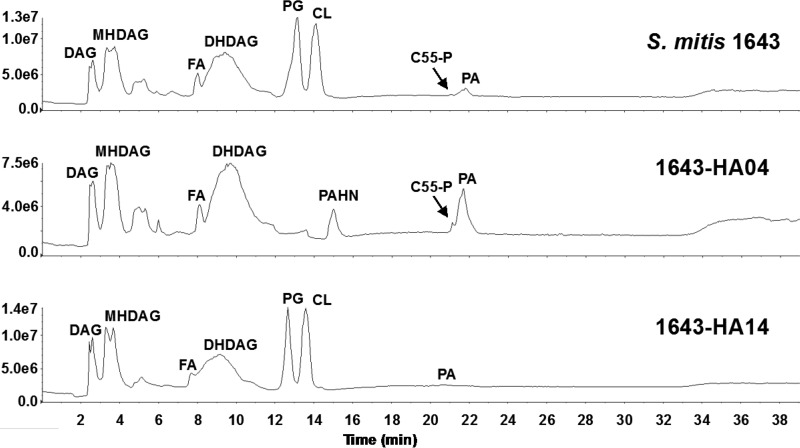
Normal-phase LC-ESI/MS analysis of the total lipid extracts of DAP-susceptible and DAP-resistant strains of S. mitis. The *y*-axis numbers represent ion intensities of arbitrary units. PG and CL are major lipids found in S. mitis 1643. PG and CL are absent in the DAP-resistant derivative 1643-HA04. After passage without selection, DAP susceptibility was restored in 1643-HA14, as were PG and CL levels.

**FIG 3:**
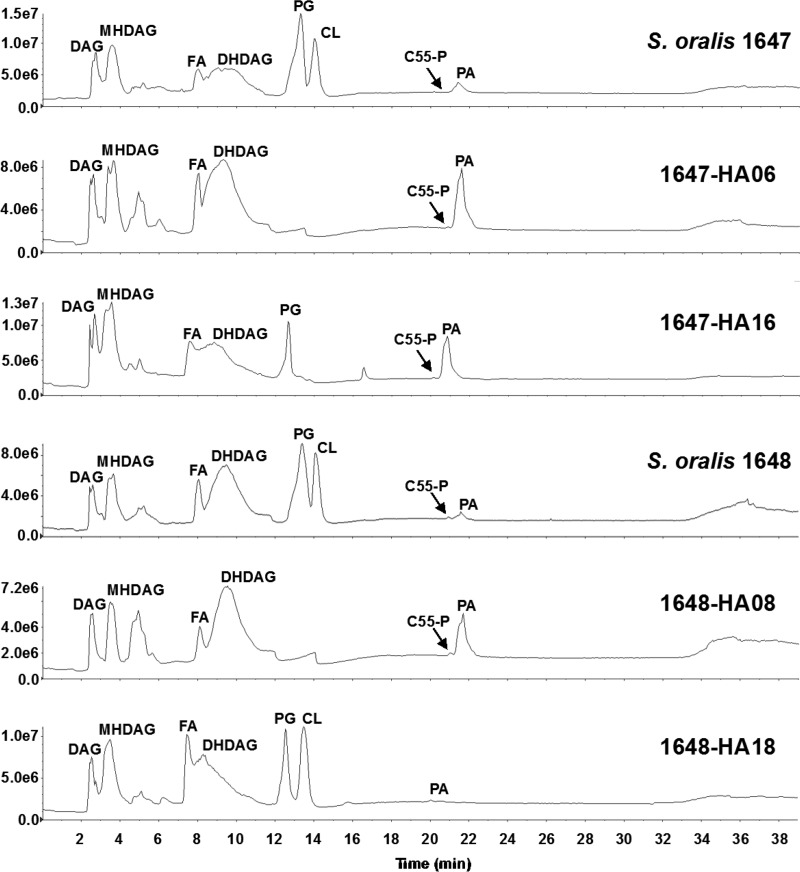
Normal-phase LC-ESI/MS analysis of the total lipid extracts of DAP-susceptible and DAP-resistant strains of S. oralis. The *y*-axis numbers represent ion intensities of arbitrary units. PG and CL are major lipids found in DAP-susceptible S. oralis 1647 and 1648. PG and CL are missing from DAP-resistant derivatives 1647-HA06 and 1648-HA08. Upon restoration of DAP susceptibility in 1647-HA16 and 1648-HA18, PG levels return to normal. CL levels are restored in 1648-HA18 but not 1647-HA16.

To assess the functional consequences of *cdsA* mutations on the synthesis of phospholipids in S. mitis and S. oralis, we carried out lipidomic analysis. The total lipids from DAP-susceptible and DAP-resistant strains of *S. mitis* and S. oralis were subjected to normal-phase liquid chromatography-electrospray ionization mass spectrometry (LC-ESI/MS) using a silica column for lipid separation. As shown by the total negative-ion chromatograms (Fig. 2 and 3; see also Fig. S2), PG and CL are the two major anionic phospholipids detected in all three DAP-susceptible strains. They appear at the retention time windows of ∼12.5 to 13.5 min and 13.5 to 14.5 min, respectively. In contrast, PG and CL are completely absent in three DAP-resistant strains. The results confirm *cdsA* inactivation in the DAP-resistant strains as well as the essential role of *cdsA* for PG and CL synthesis in *S. mitis* and S. oralis under the conditions tested.

Other species identified in both DAP-susceptible and DAP-resistant strains are diacylglycerol (DAG), monohexosyldiacylglycerol (MHDAG), fatty acid (FA), dihexosyldiacylglycerol (DHDAG), undecaprenyl phosphate (C55-P), and PA. The levels of PA are consistently increased in all DAP-resistant strains (Fig. 2 and 3). PA is a substrate of CdsA for the synthesis of CDP-DAG (Fig. 1); thus, its accumulation is an expected consequence of the inactivation of *cdsA*. The acyl compositions of PA in the DAP-sensitive strains are slightly different from those in the DAP-resistant strains (Fig. S3). For reference, the total lipid content of S. mitis 1643 and its derivatives is displayed in Table S3 in the supplemental material.

An unknown species, appearing at 14.8 to 15.3 min, is significantly elevated in the DAP-resistant S. mitis 1643-HA04. Exact mass measurement and collision-induced dissociation (CID) tandem MS identified this unknown lipid as phosphatidyl-*N*-acetylhexosamine (PAHN), a PA-derived glycolipid (Fig. S4). A small amount of PAHN was detected in the DAP-sensitive strain S. mitis 1643-RA03 but was not detected in any of the S. oralis strains.

Phosphatidylcholines (PC) were detected by positive ion ESI/MS in all strains used in this study. Shown in Fig. S3 are representative mass spectra of PCs in S. mitis 1643 and 1643-HA04. The level of PC is lower (3- to 10-fold) in the DAP-resistant strains than in the DAP-sensitive strains (Fig. S5).

### Mutations in *cdsA* revert concurrently with phenotypic reversion to DAP susceptibility.

Resistant strains were passaged in CA-SMHB until DAP susceptibility was restored. S. mitis 1643-HA04 began showing a zone of inhibition, indicating reversion, after the 4th passage, while S. oralis 1647-HA06 and S. oralis 1648-HA08 reverted after the 12th passage. Revertant populations recovered from the 5th overnight for S. mitis 1643-HA04 and the 13th overnight for S. oralis 1647-HA06 and S. oralis 1648-HA08 are referred to as S. mitis 1643-HA14, S. oralis 1647-HA16, and S. oralis 1648-HA18, respectively. These populations have MICs identical or comparable to those of the original susceptible parental strains (Table 1) and growth rates similar to those of the parental strains (Fig. S1).

Mutations listed in Table 2 were queried in the revertant populations using PCR and Sanger sequencing. All mutations present in the DAP-resistant strains were also present in the revertant populations, except for the *cdsA* mutations. Analysis of *cdsA* showed that the premature stop codon in S. mitis 1643-HA04 was replaced with a tryptophan codon in S. mitis 1643-HA14, allowing for proper read-through of the coding region; the C246 of S. oralis 1647-HA06 was replaced with a serine in S. oralis 1647-HA16, restoring flexibility to the region, and the active site of S. oralis 1648-HA08 was restored in S. oralis 1648-HA18, as the mutation reverted to wild type. Table 1 displays the DAP MICs for the susceptible, resistant, and revertant populations of each lineage, as well as the corresponding amino acid changes in CdsA. Our data indicate that restoration of CdsA function results in DAP susceptibility.

To confirm CdsA functionality in our revertants, we subjected them to lipidomic analyses as described above. All three revertant strains synthesize PG, indicating the synthesis of CDP-DAG and therefore confirming the restored function of CdsA (Fig. 2 and 3). Interestingly, 1647-HA16 does not possess any CL and continues to have a small accumulation of PA (Fig. 3). It is possible that 1647-HA16 acquired a loss-of-function mutation in a cardiolipin synthase gene over the course of the reversion passage, thereby preventing formation of CL. Alternatively, and not mutually exclusively, 1647-HA16 might possess only a partial restoration of CdsA function, resulting in an accumulation of PA. That 1647-HA16 is DAP sensitive indicates that PG, and not CL, is required for DAP susceptibility in S. oralis.

### Spontaneous DAP resistance is always associated with *cdsA* mutation.

**FIG 4:**
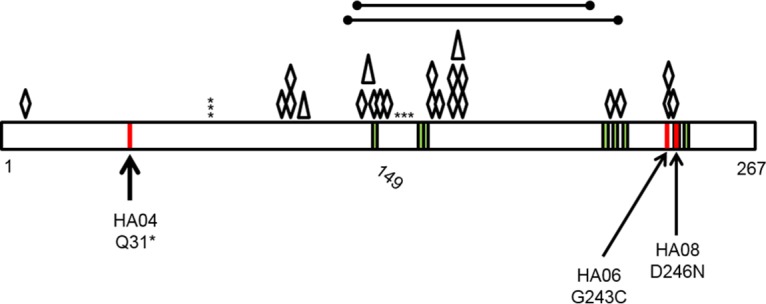
Summary of CdsA alterations identified in S. mitis and S. oralis after DAP selection. CdsA is depicted as a black rectangle, with amino acid positions indicated at the bottom. Green stripes represent active sites identified previously in Thermotoga maritima (31). Red stripes represent the original mutations identified in the broth-derived DAP-resistant strains. Asterisks depict premature stop codons, diamonds depict amino acid substitutions, triangles depict a nucleotide coding region deletion event of 1 to 2 bp, and the large lines at the top depict large areas of deletion identified in spontaneous-resistance studies on DAP agar. HA04, S. mitis 1643-HA04; HA06, S. oralis 1647-HA06; HA08, S. oralis 1648-HA08.

To assess the frequency and diversity of *cdsA* mutations, we plated S. mitis and S. oralis parental strains on DAP-containing agar and screened colonies for *cdsA* mutation. In addition, we compared the frequency of spontaneous DAP resistance to that of spontaneous resistance to rifampin, a commonly used antibiotic for mutation frequency studies. S. oralis 1648 was not analyzed due to its preexisting rifampin resistance (17). Spontaneous DAP resistance arose at an average frequency of 1 × 10^−6^ for both strains on 10 μg/ml of DAP and at an average frequency of 7 × 10^−7^ for both strains on 128 μg/ml of DAP. Rifampin resistance arose at frequencies of 1 × 10^−7^ for S. mitis 1643 and 8 × 10^−9^ for S. oralis 1647. Of colonies arising on the 10-μg/ml and 128-μg/ml DAP plates that were screened (*n* = 15 and 14, respectively), all possessed nonsynonymous mutations in *cdsA*. In two colonies, two separate polymorphisms were found. Polymorphisms were observed that resulted in amino acid substitutions (*n* = 20; 15 unique), premature stop codons (*n* = 6; 4 unique), large-scale (>200-bp) deletions (*n* = 2), or frameshifts due to small insertions or deletions (*n* = 3). The two large deletions (268 bp and 252 bp) encompassed approximately the same region, but only one of the deletions resulted in a frameshift. Figure 4 and Table S4 in the supplemental material catalog all mutations detected in *cdsA* in this study. We conclude that diverse *cdsA* mutants rapidly emerge in S. mitis and S. oralis populations under DAP selection.

## DISCUSSION

CdsA catalyzes the synthesis of CDP-DAG, a key intermediate in phospholipid biosynthesis in all cells. CDP-DAG is the source of the phosphatidyl group for all major phospholipids in prokaryotes and for most anionic phospholipids in eukaryotes. Historical efforts to elucidate the biochemical pathways for membrane phospholipid biosynthesis used chemical mutagenesis to isolate E. coli mutants with reduced CDP-DAG synthase activity (33, 34). Those mutants were observed to accumulate PA but were not complete-loss-of-function mutants due to the continued presence of PG, CL, and PE in the E. coli membrane (33, 34). More recently, E. coli partial-loss-of-function *cdsA* mutants were found to accumulate PA and have decreased vancomycin susceptibility, presumably by altering outer membrane structure and access of the drug to the periplasm (35). Efforts to define the essential gene cohorts of E. coli, B. subtilis, S. pneumoniae, and other bacteria have identified *cdsA* as being essential (23–29). Crucially, efforts to define the minimum cohort of genes required to sustain cellular life also identified *cdsA* as being essential (30). In contrast to these observations, S. mitis 1643-HA04, S. oralis 1647-HA06, and S. oralis 1648-HA08 lack CdsA function, as confirmed by lipidomics analysis. Furthermore, lipidomics results demonstrate that a CdsA-independent pathway for anionic phospholipid biosynthesis does not exist or is not active in these bacteria under the conditions tested in this study.

Why is *cdsA* essential in other bacteria but not in S. mitis and S. oralis? Presumably, *cdsA* is essential in other bacteria because the lipids synthesized from the CdsA product, CDP-DAG, are essential for cytoplasmic membrane integrity, fluidity, and/or the localization of key proteins for cellular maintenance and replication. Alternatively, the substrate of CdsA, phosphatidic acid, could be toxic at high levels. Potential physiological mechanisms for growth of S. mitis and S. oralis cdsA mutants include altered cell wall structure and/or altered distribution or perhaps a lack of necessity for key membrane proteins involved in cell division. These mechanisms would be expected to be absent from model bacteria for which *cdsA* is essential. Unfortunately, little is known about the physiology and genetics of S. mitis and S. oralis. Characterization of peptidoglycan structure, membrane proteomics, and the transcriptomes of the wild type and *cdsA* mutants will be informative to understand the mechanism of survival of S. mitis and S. oralis cdsA mutants. In addition, studies which assess the impact of altered membrane structure and altered growth rates on the virulence and *in vivo* physiology of DAP-resistant S. mitis and S. oralis are warranted.

PC is a relatively rare membrane phospholipid in the bacterial domain, with only ∼15% of bacteria encoding the necessary biosynthetic pathway (36). Eukaryotes synthesize PC using the Kennedy pathway (37). A homologous pathway has been discovered in the bacterium Treponema denticola (38, 39). The first two genes in the pathway are *licA* and *licC*. BioCyc is a pathway and genome database which predicts metabolic networks for various organisms (40). S. mitis and S. oralis are predicted to possess homologs of the first two genes in the Kennedy pathway; however, the third gene has yet to be elucidated. S. pneumoniae uses *licA* and *licC* to incorporate choline into the cell wall to aid in adhesion to human epithelial cells (41). It is possible that the *cdsA* mutants repurpose the *lic* pathway for PC synthesis under certain conditions. Other Gram-positive model organisms, such as Enterococcus faecalis, S. aureus, and B. subtilis, do not carry *licA* and *licC*. This is further evidence of S. mitis and S. oralis possessing unique physiological characteristics that allow them to tolerate CdsA loss of function.

Studies with other Gram-positive bacteria have associated a wide range of mutations with reduced DAP susceptibility. A common feature identified by some of these studies is a bacterial cell membrane with reduced PG content. Mutations in *mprF* were shown to increase lysyl-PG content in DAP-nonsusceptible MRSA, concomitantly reducing the PG content of the membrane (42). In B. subtilis, an evolved DAP-nonsusceptible strain possessed reduced function mutations in *pgsA*, which is responsible for the synthesis of PG (43). The evolved B. subtilis strain possessed PG levels >5-fold lower than its susceptible parental strain and a DAP MIC nearly 30-fold higher. In this study, we have identified a novel mechanism by which S. mitis and S. oralis purge their membranes of PG and, as previously shown, DAP MICs increase up to 512-fold higher than DAP-sensitive parental strains (17, 18).

Several models for DAP's mechanism of action against Gram-positive bacteria have been proposed (21, 22). These mechanisms generally implicate membrane pore formation by DAP or recruitment of DAP to sites of membrane curvature. More recently, a revised mechanism of DAP action was proposed for the model Gram-positive bacterium B. subtilis (44). In this model, DAP's lipid tail interacts with domains of increased membrane fluidity, allowing DAP to oligomerize at those sites. This causes rapid delocalization of membrane proteins essential for peptidoglycan biosynthesis (MurG) and lipid biosynthesis (PlsX), which are also associated with the membrane fluid domains. The displacement of MurG interrupts peptidoglycan biosynthesis, ultimately leading to cell lysis. DAP-resistant S. mitis and S. oralis may synthesize a membrane for which DAP has low affinity, and loss of CdsA activity is required for synthesis of this membrane. Our data indicate that PG is a critical phospholipid associated with DAP susceptibility. Alternatively, S. mitis and S. oralis may not organize essential membrane proteins in membrane fluid domains. The biophysical mechanism for DAP resistance in these strains remains to be determined. It will be interesting to determine whether S. mitis and S. oralis *cdsA* mutants emerge in humans treated with DAP and whether these *cdsA* mutations are as permissive for S. mitis and S. oralis outgrowth under human *in vivo* conditions as they are under *in vitro* conditions.

## MATERIALS AND METHODS

### Bacterial strains, media, and susceptibility testing.

Bacterial strains used in this study are shown in Table 1. All testing was conducted in Mueller-Hinton broth or agar supplemented with 50 μg/ml of calcium and 12.5 μg/ml of magnesium (CA-SMHB and CA-SMHA, respectively) as required for DAP (45, 46), unless otherwise stated. Cultures were incubated at 37°C in a GasPak EZ Campy container system (BD) unless otherwise stated. Etest (bioMérieux, Inc.) susceptibilities were determined on tryptic soy agar (TSA) supplemented with 5% defibrinated horse blood (Remel) or on MHA.

### Serial passage experiments with DAP.

Parental strains (S. mitis 1643, S. oralis 1647, and S. oralis 1648) were cultured in 2 ml of CA-SMHB with DAP (2, 4, 8, and 16 μg/ml) overnight at 37°C. After 24 h of incubation, growth was observed in each inoculated well, regardless of the DAP concentration. Strains obtained from these cultures (referred to as HA0X [Table 1]) were confirmed to be DAP resistant and used for genome sequencing.

### Genome sequencing and analysis.

Genomic DNA from parental strains was isolated using Roche MagNA Pure per the manufacturer's instructions and sequenced with Illumina technology at GENEWIZ, Inc. Single end reads of 50 bp were obtained. Genomic DNA from resistant strains was isolated using a modified Qiagen blood and tissue DNeasy kit protocol as previously described (47) and sequenced with Illumina technology at Molecular Research LP. Paired end, 2 × 300-bp reads were obtained. DAP resistance of the cultures was confirmed via Etest prior to genomic DNA extraction.

*De novo* draft genomes were assembled using CLC Genomics Workbench with default parameters (Table S1) and annotated using Rapid Annotation using Subsystem Technology (48). The taxonomic identification for the parental strains were confirmed using GyrB typing (49). Reads from the resistant strains were mapped to the draft parent genomes, and polymorphisms were detected using default parameters. Polymorphisms were manually curated. Polymorphisms occurring on contigs of <500 bp, within 300 bp from a contig end, in rRNA or tRNA regions, or in polymorphic regions with sequence variation in both the parental *de novo* assembly and the resistant strain read mapping were removed from further analysis. This screening process generated a list of candidate mutations that were further curated by independent confirmation with Sanger sequencing. Primers were designed to amplify approximately 500 bp surrounding putative mutations (Table S2). PCR with *Taq* polymerase (New England BioLabs [NEB]) was used to amplify these regions from both parental and resistant strains. Products were sequenced at the Massachusetts General Hospital DNA Sequencing Core. Nucleotide sequences for wild-type and mutant loci of interest are in Text S1 in the supplemental material.

### Bacterial growth curves.

To quantify growth, brain heart infusion broth (BHI) was inoculated to an optical density at 600 nm (OD_600_) of 0.05 from overnight cultures. The OD_600_ was monitored for parental and resistant strains every 60 to 75 min until cultures reached stationary phase. The experiments were performed independently three times. Revertant strains were monitored as described above at 60-min intervals for two independent trials.

### Reversion passage experiments.

Resistant strains (S. mitis 1643-HA04, S. oralis 1647-HA06, and S. oralis 1648-HA08) were inoculated into 1 ml of CA-SMHB in 1.5-ml Eppendorf tubes and cultured overnight. After each passage, 100 μl of culture was used as the inoculum for the next passage, 500 μl was used to generate a frozen stock, and ∼400 μl was spread on MHA for DAP Etest. Passaging was terminated when a zone of inhibition was observed on DAP Etest.

### Spontaneous resistance incidence.

Overnight cultures were used to inoculate BHI to an OD_600_ of 0.05. Cultures were incubated until mid-exponential phase and serially diluted, and 100 μl of culture or dilutions were plated on BHI agar, BHI agar with 50 μg/ml of rifampin (*n* = 2 independent trials), CA-SMHA, and CA-SMHA with either 10 μg/ml of DAP (*n* = 5 independent trials) or 128 μg/ml of DAP (*n* = 3 independent trials). Plates were incubated for 20 to 24 h at 37°C in a chamber with a CO_2_ GasPak prior to colony counting. For DAP tests, individual colonies were picked, and PCR and Sanger sequencing (Table S2) were used to query the entire *cdsA* coding region as well as its putative promoter for mutations.

### Lipidomic analysis.

A single colony was picked into 50 ml of Todd-Hewitt broth (THB) and incubated at 37°C with 5% CO_2_ overnight. The 50-ml cultures were added to 250 ml of prewarmed THB and incubated until an OD_600_ of ∼0.6 was obtained. A total of 500 μl was removed for DAP Etest on 5% horse blood TSA plates. The remaining culture was pelleted at 10,000 rpm and 4°C. Cell pellets were stored at −80°C prior to lipid extraction by the Bligh and Dyer method (50). Normal-phase LC was performed on an Agilent 1200 quaternary LC system equipped with an Ascentis silica high-performance liquid chromatography (HPLC) column (5 μm; 25 cm by 2.1 mm; Sigma-Aldrich) as published previously (51, 52). Briefly, mobile phase A consisted of chloroform-methanol-aqueous ammonium hydroxide (800:195:5, vol/vol), mobile phase B consisted of chloroform-methanol-water-aqueous ammonium hydroxide (600:340:50:5, vol/vol), and mobile phase C consisted of chloroform-methanol-water-aqueous ammonium hydroxide (450:450:95:5, vol/vol/vol/vol). The elution program consisted of the following: 100% mobile phase A was held isocratically for 2 min, then linearly increased to 100% mobile phase B over 14 min, and held at 100% mobile phase B for 11 min. The LC gradient was then changed to 100% mobile phase C over 3 min, held at 100% mobile phase C for 3 min, and, finally, returned to 100% mobile phase A over 0.5 min and held at 100% mobile phase A for 5 min. The LC eluent (with a total flow rate of 300 ml/min) was introduced into the ESI source of a high-resolution TripleTOF5600 mass spectrometer (Sciex, Framingham, MA). Instrumental settings for negative-ion ESI and MS/MS analysis of lipid species were as follows: IS = −4,500 V, CUR = 20 lb/in^2^, GSI = 20 lb/in^2^, DP = −55 V, and FP = −150 V. The MS/MS analysis used nitrogen as the collision gas. Data analysis was performed using Analyst TF1.5 software (Sciex).

### Accession number(s).

Illumina sequence reads generated in this study have been deposited in the Sequence Read Archive under the accession number PRJNA354070.

## Supplementary Material

Supplemental material
