# Supplementary material for: Streptococcus mitis and S. oralis Lack a Requirement for CdsA, the Enzyme Required for Synthesis of Major Membrane Phospholipids in Bacteria
Source: Antimicrob Agents Chemother. 2017 Apr 24;61(5):e02552-16. doi: 10.1128/AAC.02552-16 (PMC5404519; doi:10.1128/AAC.02552-16)
Supplement: Supplemental material [file supp_61_5_e02552-16__index.html]

Streptococcus mitis and S. oralis Lack a Requirement for CdsA, the Enzyme Required for Synthesis of Major Membrane Phospholipids in Bacteria — Supplemental material 

# Streptococcus mitis and S. oralis Lack a Requirement for CdsA, the Enzyme Required for Synthesis of Major Membrane Phospholipids in Bacteria

## Supplemental material

- Supplemental file 1 -

  Supplemental Figures S1 to S5, Tables S1 to S4, and Text S1

  PDF, 1.1M
