# Supplementary material for: Streptococcus mitis and S. oralis Lack a Requirement for CdsA, the Enzyme Required for Synthesis of Major Membrane Phospholipids in Bacteria
Source: Antimicrob Agents Chemother. 2017 Apr 24;61(5):e02552-16. doi: 10.1128/AAC.02552-16 (PMC5404519; doi:10.1128/AAC.02552-16)
Supplement: Supplemental material [file AAC.02552-16_zac005176127s1.pdf]

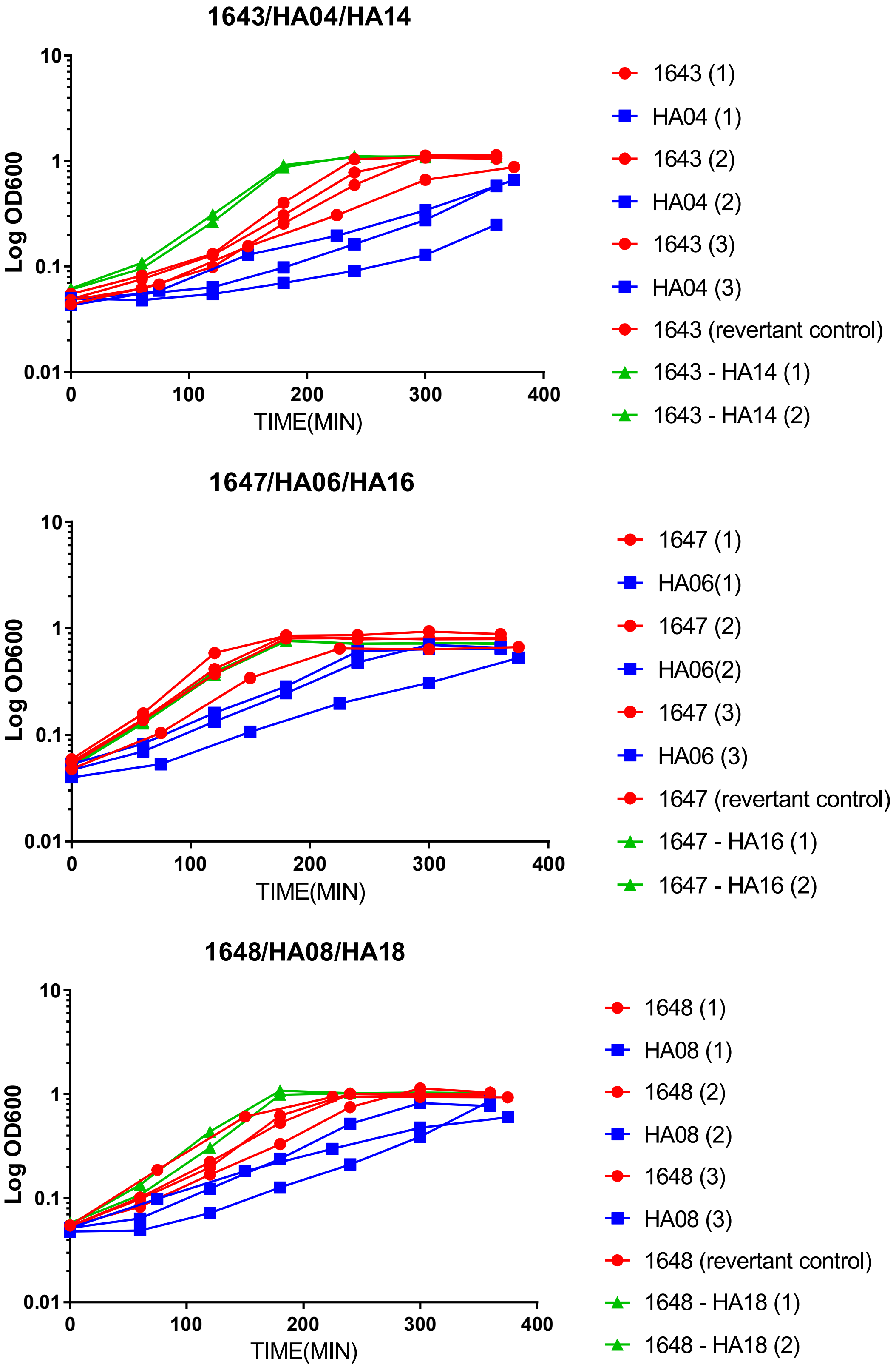

Figure S1. Growth curves for parental strains, their DAP^R derivatives, and DAP^S revertants. *S. mitis* 1643, DAP^R derivative 1643-HA04, and DAP^S revertant 1643-HA14 are displayed top; *S. oralis* 1647, DAP^R derivative 1647-HA06, and DAP^S revertant 1647-HA16 are displayed middle; *S. oralis* 1648, DAP^R derivative 1648-HA08, and DAP^S revertant 1648-HA18 are shown bottom. Red lines represent parental strains, blue lines represent resistant strains, and green lines represent revertant strains. Cultures were grown overnight in BHI and diluted to an OD_{600} of 0.05 in fresh BHI. OD_{600} readings were taken either every hour or every 75 minutes for six hours.

Figure S2: Representative negative ion ESI mass spectra of PG and CL species in a DAP sensitive/resistant strain pair.

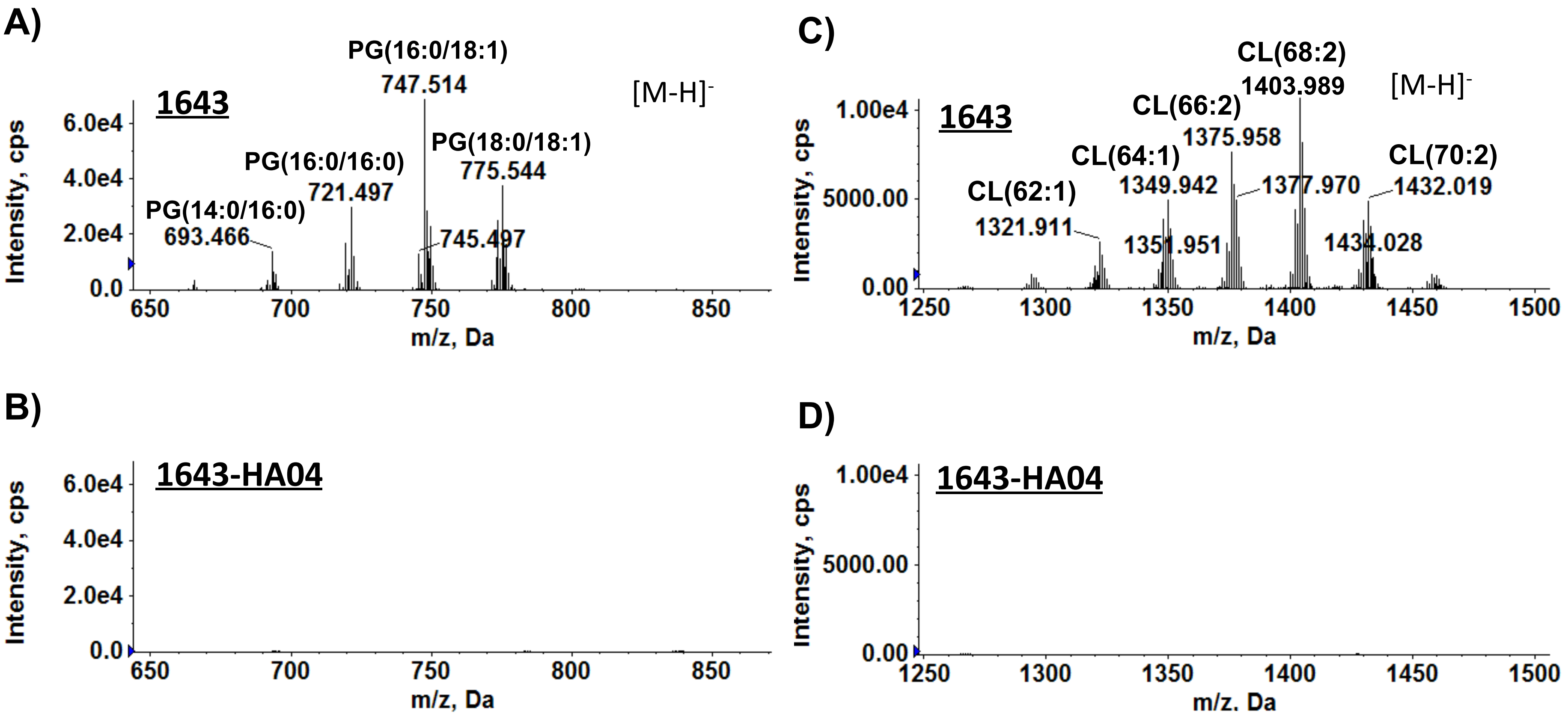

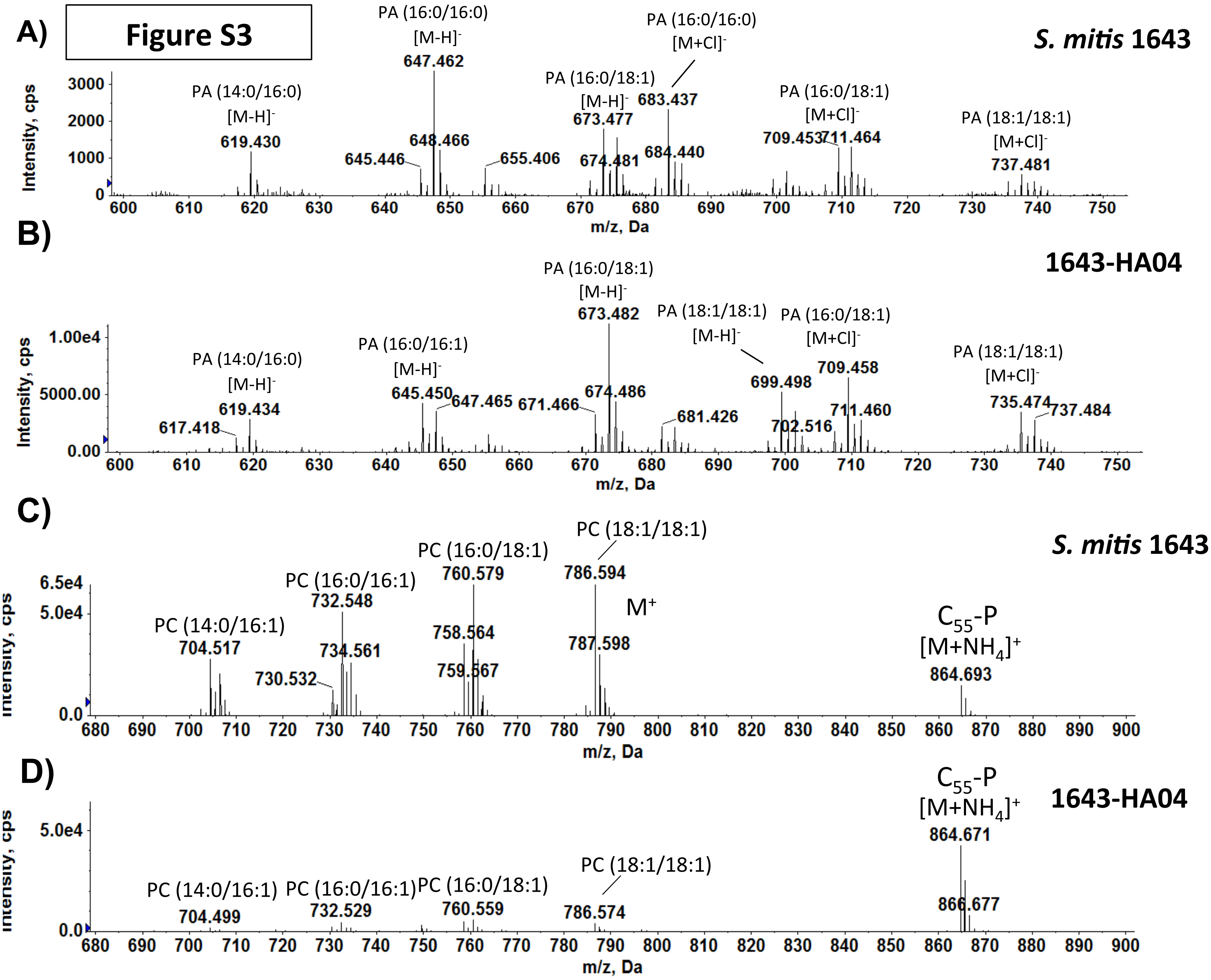

Figure S3
A)
S. mitis 1643
PA (14:0/16:0) [M-H]- 619.430
PA (16:0/16:0) [M-H]- 647.462
645.446
648.466
655.406
PA (16:0/18:1) [M-H]- 673.477
674.481
PA (16:0/16:0) [M+Cl]- 683.437
684.440
PA (16:0/18:1) [M+Cl]- 709.453 711.464
PA (18:1/18:1) [M+Cl]- 737.481
Intensity, cps
m/z, Da
B)
1643-HA04
PA (14:0/16:0) [M-H]- 619.434
617.418
PA (16:0/16:1) [M-H]- 645.450
647.465
671.466
PA (16:0/18:1) [M-H]- 673.482
674.486
681.426
PA (18:1/18:1) [M-H]- 699.498
702.516
PA (16:0/18:1) [M+Cl]- 709.458
711.460
PA (18:1/18:1) [M+Cl]- 735.474
737.484
Intensity, cps
m/z, Da
C)
S. mitis 1643
PC (14:0/16:1) 704.517
PC (16:0/16:1) 732.548
730.532
734.561
PC (16:0/18:1) 760.579
758.564
759.567
PC (18:1/18:1) 786.594
787.598
M+
C55-P [M+NH4]+ 864.693
Intensity, cps
m/z, Da
D)
1643-HA04
PC (14:0/16:1) 704.499
PC (16:0/16:1) 732.529
PC (16:0/18:1) 760.559
PC (18:1/18:1) 786.574
C55-P [M+NH4]+ 864.671
866.677
Intensity, cps
m/z, Da

Fig S3. Mass spectra of PAs, PCs, and undecaprenyl phosphate (C_{55}-P) in (A), (C) DAP-sensitive and (B), (D) DAP-resistant strains. The mass spectra shown are averaged from spectra acquired by negative ion ESI/MS during the 20.2-21.2 min window. Both deprotonated $[M-H]^-$ and chloride adduct $[M+Cl]^-$ ions of PA are detected. The level of PC is significantly lower in the DAP-resistant strain compared to the parental DAP-sensitive strain.

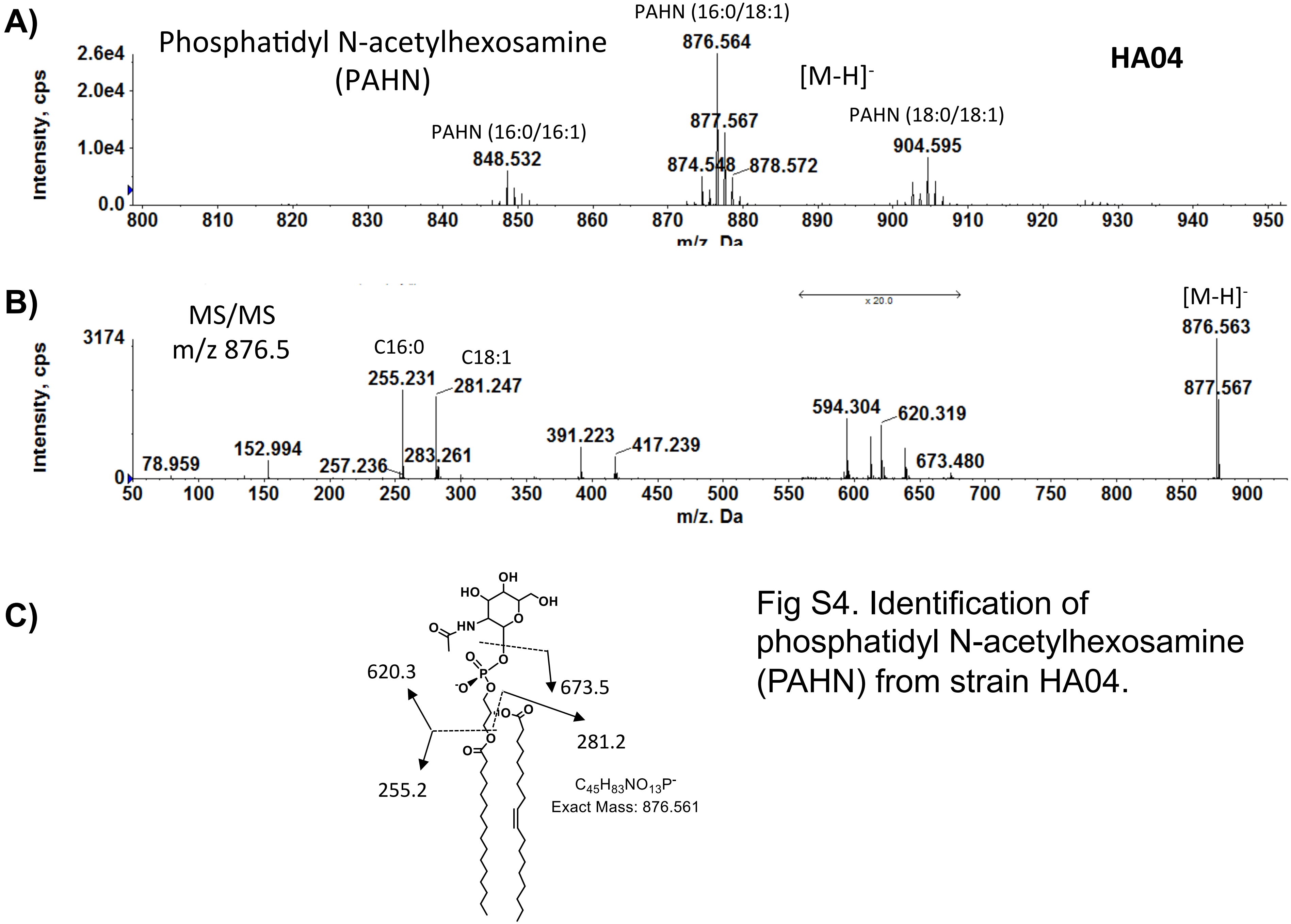

Fig S4. Identification of phosphatidyl N-acetylhexosamine (PAHN) from strain HA04.

Fig S4. Identification of phosphatidyl N-acetylhexosamine (PAHN) from strain HA04.

A. Negative ion ESI mass spectrum showing the $[M-H]^-$ ions of the PAHN molecular species emerging at 14.8-15.3 min.
B. Collision-induced dissociation (CID) MS/MS spectrum of the $[M-H]^-$ ion PAHN (16:0/18:1) at *m/z* 876.5. The arrows indicating x20 reflect magnification of the product ion peaks in the corresponding region of the *m/z* values on the mass spectrum.
C. Chemical structure of PAHN (16:0/18:1) and the fragmentation scheme for the observed product ions in B.

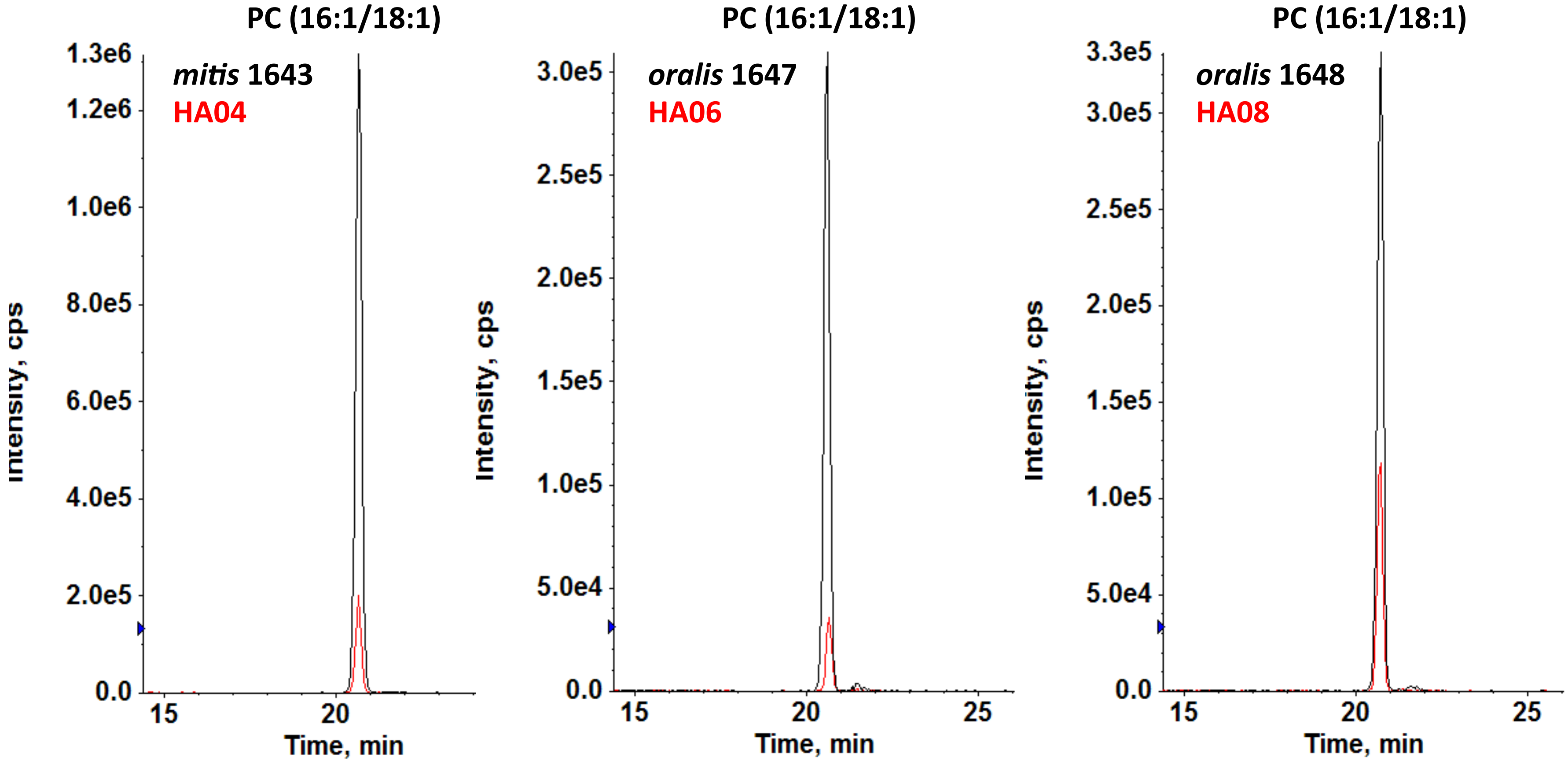

Fig S5. Comparison of the level of PC (16:1/18:1) in the DAP-resistant and DAP-sensitive pair strains. Extracted ion chromatograms of *m/z* 758.5 for the M^+ ion of PC (16:1/18:1), a major PC species in *S. mitis* 1643 (black) and *S. mitis* 1643-HA04 (red).

Table S1. De novo assembly data for parental strains.

Strain	# reads	# contigs	Min contig size	Max contig size	Average contig size	Contig N50 (bp)	Genome Size (Mbp)
1643	12,173,871	119	211	253,142	15,805	66,153	1,880,744
1647	14,577,054	158	202	224,156	12,380	53,890	1,956,052
1648	14,503,619	199	205	153,663	9,413	48,229	1,873,268

Table S2. Primers used in this study.

Primer Name	Sequence
1643_IntGen_19_F	CCAGTGTAACCTTTACTGCA
1643_IntGen_19_R	CGAGCTGACTGAAGCAGAC
1643_CellWall_F	GCCTGACTTAACTAATCTGG
1643_CellWall_R	CCACGAAGTCGAATCGTTAC
1643_Hypothetical_F	GGAGATTTAGAGGGGTGTC
1643_Hypothetical_R	CGGAACTATATCCAATCTGTC
1643_SerThr_Kinase_F	GCGTCAGATAAGTGAAGTGG
1643_SerThr_Kinase_R	CCCATCCACGAAGAAAACAT
1643_IntGen_32_F	GTGCCGTCAGTTTTGACTTG
1643_IntGen_32_R	GGACTACTGTAGCTGACAC
1643_Ribo_S5p_F	GCCAGATGGCGTCAAAGT
1643_Ribo_S5p_R	GGCTAGTGATGTCGGAGAG
1643_cdsA_F	GCAATTGGGAAAGCGGCA
1643_cdsA_R	CCGTACGAGTGGTGAGTTG
1647_IntGen_13_F	GCTACAAAAGCGCAGGCTTG
1647_IntGen_13_R	GGCTTACTATGGCTTTGAC
1647_DAG_Kinase_F	CGGCTCCTGCCGCCATGT
1647_DAG_Kinase_R	GCCTACATTGGGGAACTTTTC
1647_IntGen_17_F	CGAAATCAGATAGACTTGTCC
1647_IntGen_17_R	GATTTGGCTCTTTTACCATAC
1647_cdsA_F	GGGCCCCATACCAATGGC
1647_cdsA_R	GCGCAGCCTACCTGACAG
1647_Ribo_Mtase_F	CCAGCAGTCGCCTCATAG
1647_Ribo_Mtase_R	GCCCTAAACACTGTGCCA
1647_ppGpp_Synthetase_F	GGACTATGCTACCGAACG
1647_ppGpp_Synthetase_R	CTCCTCCGCCAGATAGTT
1648_cdsA_F	GAGGAAGGATTCGAATGGT
1648_cdsA_R	GGCTCCGAGAGTTTCTCC
1648_IntGen_27_F	GCTCACCAATCCCAAACAC
1648_IntGen_27_R	GGAACGCGTTCTCAGAAAG
1648_ppGpp_Synthetase_F	CCTCTGGGCCAACACCG
1648_ppGpp_Synthetase_R	GCAATCCAGTGCCGGGTG
1648_Ribo_SSU_Mtase_F	CCAGTTGACTAGACTCGTC
1648_Ribo_SSU_Mtase_R	CGAAGACAGAGATAAGCTAG
1648_Helicase_F	GGAAGCATGGCTTTTAGGATG
1648_Helicase_R	CGTGAAGCGGTTCGTCTC
Full_cdsA_F	GGTTAGCAATCCAATCATTC
Full_cdsA_R	CCATCGCAATCGCCGTTTTG
CdsA_promoter_F	GCCTCCTAGAGGAAGGAT
CdsA_promoter_R	GGGACGGTATACTAGGTTC
CdsA_Sequence_1	GCCTTGCAGGAAGCTATTG

Table S3. Major lipid species in wild-type, *cdsA* mutant, and revertant *S. mitis* detected by negative ion ESI/MS.

Lipid class	$[M-H]^-$	Molecular species [a]	WT	CdsA	Revertant
DAG	547.42[b]	DAG (26:0)	+	+	+
	573.43[b]	DAG (28:1)	+	+	+
	575.44[b]	DAG (28:0)	+	+	+
	601.46[b]	DAG (30:1)	+	+	+
	603.47[b]	DAG (30:0)	+	+	+
	627.47[b]	DAG (32:2)	+	+	+
	629.49[b]	DAG (32:1)	+	+	+
	657.52[b]	DAG (34:1)	+	+	+
FA	225.19	FA (14:1)	+	+	+
	227.2	FA(14:0)	+	+	+
	253.22	FA (16:1)	+	+	+
	255.23	FA (16:0)	+	+	+
	281.25	FA (18:1)	+	+	+
	283.26	FA (18:0)	+	+	+
MHDAG	709.46[b]	MHDAG (26:0)	+	+	+
	735.48[b]	MHDAG (28:1)	+	+	+
	737.49[b]	MHDAG (28:0)	+	+	+
	763.50[b]	MHDAG (30:1)	+	+	+
	765.51[b]	MHDAG (30:0)	+	+	+
	791.54[b]	MHDAG (32:1)	+	+	+
	819.57[b]	MHDAG (34:1)	+	+	+
DHDAG	871.52[b]	DHDAG (26:0)	+	+	+
	897.53[b]	DHDAG (28:1)	+	+	+
	899.54[b]	DHDAG (28:0)	+	+	+
	925.56[b]	DHDAG (30:1)	+	+	+
	927.57[b]	DHDAG (30:0)	+	+	+
	953.58[b]	DHDAG (32:1)	+	+	+
	981.62[b]	DHDAG (34:1)	+	+	+

PG	693.47	PG(14:0/16:0)	+	—	+
	719.49	PG(16:0/16:1)	+	—	+
	721.5	PG(16:0/16:0)	+	—	+
	773.53	PG(16:0/18:1)	+	—	+
	775.54	PG(18:0/18:1)	+	—	+
CL	1321.91	CL(62:1)	+	—	+
	1349.94	CL(64:1)	+	—	+
	1375.96	CL(66:2)	+	—	+
	1403.99	CL(68:2)	+	—	+
	1432.02	CL(70:2)	+	—	+
PAHN	848.53	PAHN(16:0/16:1)	+	+	+
	874.55	PAHN(16:1/18:1)	+	+	+
	876.56	PAHN(16:0/18:1)	+	+	+
	902.58	PAHN(18:1/18:1)	+	+	+
	904.6	PAHN(18:0/18:1)	+	+	+
PA	619.43	PA(14:0/16:0)	+	+	+
	645.45	PA(16:0/16:1)	+	+	+
	647.46	PA(16:0/16:0)	+	+	+
	673.48	PA(16:0/18:1)	+	+	+
	699.49	PA(18:1/18:1)	+	+	+
	701.50	PA(18:0/18:1)	+	+	+
PC	740.50[b]	PC(14:0/16:0)	+	+	+
	766.51[b]	PC(16:0/16:1)	+	+	+
	792.53[b]	PC(16:1/18:1)	+	+	+
	794.54[b]	PC(16:0/18:1)	+	+	+
	820.56[b]	PC(18:1/18:1)	+	+	+

[a]The individual or total numbers of acyl chain carbon atoms and double bonds are shown in parentheses.
[b]DAG, MHDAG, DHDAG, and PC values are for the $[M + Cl]^-$ ions.
Abbreviations: WT, *S. mitis* 1643; CdsA, *S. mitis* 1643-HA04; Revertant, *S. mitis* 1643-HA14; DAG, diacylglycerol; MHDAG, monohexosyldiacylglycerol; DHDAG, dihexosyldiacylglycerol; FA, fatty acid; CL, cardiolipin; PA, phosphatidic acid; PAHN, phosphatidyl N-acetylhexosamine; PE, phosphatidylethanolamine; PG, phosphatidylglycerol; PC, phosphatidylcholine.

Table S4. Mutations in *cdsA* identified in daptomycin spontaneous resistance experiments.

Strain ID	Nucleotide Change	Amino Acid Change
LJ35	G35A	A12E
LJ39	T264A	Y88*
LJ310	T264A	Y88*
LJ311	T264A	Y88*
LJ713	C351A	S117R
LJ72	G361T	G121C
LJ76	G361T	G121C
LJ313	T366del	F122fs
LJ510	G394-T645del	V132-S215 del
LJ72	G421A	A141T
LJ54	T428-T695del	F143-F232delfs
LJ34	T431ins	I144fs
LJ56	G445A	D149N
LJ514	C450G	S150R
LJ714	C455T	A152V
LJ76	C459G	Y153*
LJ52	A481T	R161*
LJ53	A499T	R167*
LJ31	C506T	S169F
LJ51	C506T	S169F
LJ712	G516C	K172N
LJ33	G527C	G176A
LJ37	G527C	G176A
LJ711	T531del	F177fs
LJ32	C551A	A184E
LJ59	C551A	A184E
LJ36	G679C	A227P
LJ512	C688T	R230C
LJ312	A731C	H244P
LJ314	A731C	H244P
LJ55	G734T	G245V

***S. mitis* 1643/RA04**

>1643_Hypothetical

MTTKNYFIRSFNLILYLVLSTPICSVFGWLFNFLSVQLANISFVNLDSVKNITDTISSVSHKSALISL
LLALLLTGLEIIQRLKTDSLRNYIQSVYHTFLLRHFLFQRERVQKISNLEHQTVTTINPIHKGFNRA
VRKCIVDIRKDTITIFLKVPRDQQGQKILKEMEAQLKEEIVSQHADYYFSSPIRVRNQLWFTGQK
R

>HA04_Hypothetical

MTTKNYFIRSFNLILYLVLSTPICSVFGWLFNFLSVQLANISFVNLDSVKNITDTISSVSHKSALISL
LLALLLTGLEIIQRLKTDSLRNYIQSVYHTFLLRHFLFQRERVQKISNLEHQTVTTINPIHKGFNRA
VRKCIVDIRKDTITIFLKVPRDQQGQKILKEMEAQLKEEIVRQHADYYFSSPIRVRNQLWFTGQK
R

>1643_Ser_Thr_Kinase

MINSDFYKRLAKIFCGDETELFTYKSGSQLVSFFNTHFHTQDSYGQGFPTRWIYVNDKLLDFSS
RGIINSFFNLILSKQYLLTERQISEVDAIEHQQKIINELDKICSVYSLKLSRKGNEFYLVEIDLDLVEI
GKGGFADIYFQKSTGLVVKKLNEESVRRQSLRSRLKREYEITKSCSDIESIIRVFDFDSSNCSYT
MEKADDTLGNYIEASELTEDSKLNILRQILYTMSLVHQRDVLHRDLSPTNVFFVDGIIKIADFGLG
KNLNTLTSHQTMDTTSFGQLFYCAPEQLSLLKDADKRSDVYSLGRIINFVMTKNPNIFSHSLRSV
SEKATNLEPDYRYQDATEMLNALNAWLSIRSGETFKKTIWEKIDHGIFDDDIENYIYEMTARELC
RACIKKSDVFIESLMVFMKLDDAHSIYIIQTIHSNYEQYLKRFEDADPFASLSYRILKGQFSFNVKE
VAAQILHYVAYEVGRFSAQRKVDNLIENGIEPLIESILER

>HA04_Ser_Thr_Kinase

MINSDFYKRLAKIFCGDETELFTYKSGSQLVSFFNTHFHTQDSYGQGFPTRWIYVNDKLLDFSS
RGIINSFFNLILSKQYLLTERQISEVDAIEHQQKIINELDKICSVYSLKLSRKGNEFYLVEIDLDLVEI
GKGGFADIYFQKSTGLVVKKLNEESVRRQSLRSRLKREYEITQSCSDIESIIRVFDFDSSNCSYT
MEKADDTLGNYIEASELTEDSKLNILRQILYTMSLVHQRDVLHRDLSPTNVFFVDGIIKIADFGLG
KNLNTLTSHQTMDTTSFGQLFYCAPEQLSLLKDADKRSDVYSLGRIINFVMTKNPNIFSHSLRSV
SEKATNLEPDYRYQDATEMLNALNAWLSIRSGETFKKTIWEKIDHGIFDDDIENYIYEMTARELC
RACIKKSDVFIESLMVFMKLDDAHSIYIIQTIHSNYEQYLKRFEDADPFASLSYRILKGQFSFNVKE
VAAQILHYVAYEVGRFSAQRKVDNLIENGIEPLIESILER

>1643_Ribosomal_alanineacyltransferase

MSLEELWQLFPIFLVEHKSEWKDWYESEKTSLKKILGANVIKRIEHIGSTAIPNIWAKNIVDILLEV
GRIEDLARVRDLLVKNGWLVMSESPNRISLNKGYTEQGFAENVFHLHLRMTGDHDEIYFRDYL
CQHPDIAQAYQELKLSLWKQFEHNRDAYTDAKTDFINHYVSEAKSSNFQESEKCHQKTLDRRK
N

>HA04_Ribosomal_alalineacyltransferase

MSLEELWQLFPIFLVEHKSEWKDWYESEKTSLKKILGANVIKRIEHIGSTAIPNIWAKNIVDILLEV
GRIEDLARVRDLLVKNGWLVMSESPNRISLNKGYTEQGFAENVFHLHLRMTGDHDEIYFRDYL
CQHPDIAQAYQELKLSLWKQFEHNRDAYTDAKTDFINNYVSEAKSSNFQESEKCHQKTLDRRK
N

>1643_cdsA

MTKDLQKRTLFAVLALAIFLPVLFAGGLLLQIGIGLLAMLGVHELLHMKGLKTMTIEGALTLFATFA
LTVPLENYLTFLPVDGNVVAYSVLITIMLGTTVFSKNYTIEDAAFPIAVSFYVGFGFNALLDARVA
GFDKVLLALFIVWATDSAAYLTGMNFGKHRLAPRVSPNKSIEGFVGGILGAVLITVIFMLVDSTVA
LPYGIYRMSLFAAFFSVAGQFGDLIESAMKRHFGVKDSGKFIPGHGGVLDRFDSMLIVFPMMHL
FGLF

>HA04_cdsA

MTKDLQKRTLFAVLALAIFLPVLFAGGLLL*

***S. oralis* 1647/RA06**

>1647_cdsA

MTKDLQKRTLFAVLALAIFLPVLFAGGLLLQIGIGLLAMLGVHELLHMKGLKTMTIEGALTLFATFA
LTVPLENYLTFLPVDGNVVAYSVLITIMLGTTVFSKNYTIEDAAFPIAVSFYVGFGFNALLDARVA
GFDKVLLALFIVWATDSAAYLTGMNFGRHKLAPRVSPNKSIEGFVGGILGAVLITVIFMLVDSTVA
LPYGIYRMSLFAAFFSVAGQFGDLIESAMKRHFGVKDSGKFIPGHGGVLDRFDSMLIVFPMMHL
FGLF

>HA06_cdsA

MTKDLQKRTLFAVLALAIFLPVLFAGGLLLQIGIGLLAMLGVHELLHMKGLKTMTIEGALTLFATFA
LTVPLENYLTFLPVDGNVVAYSVLITIMLGTTVFSKNYTIEDAAFPIAVSFYVGFGFNALLDARVA
GFDKVLLALFIVWATDSAAYLTGMNFGRHKLAPRVSPNKSIEGFVGGILGAVLITVIFMLVDSTVA
LPYGIYRMSLFAAFFSVAGQFGDLIESAMKRHFGVKDSGKFIPGHGCVLDRFDSMLIVFPMMHL
FGLF

>1647_Intergenic_Contig_13

TTATTTCTTTGACTGTGTTTCTTCTTTCTTTGTACGTGCCAATCGAAGAGCGCGTTTAGGATT
GAGACGATTAAATAATTCTTCTAGTTTCTTTTCATTCCATACATCTGCTGCAGAAACAAAATT
GCCATCTGCATCTCGGAATGATATCGGTTTATCTCCCATATCAATCTCCTAGTCTACAAATT
CAAATTCAAATTTACCAATGCGGACCAAATCGCCATCTTTGGCGCCACGCGCACGAAGGG
CTTCGTCAACTCCCATACCACGAAGCTGACGGGCAAACTTCATGACCGATTCGTCACGGTC
AAAGTTGGTCATGTTAAAGAGTTTCATGAGTTTTTCACCAGAAAGTACCCATGTCGCATCAT
CATCACGACTAATCTCAAAGGCTTTTTCTTCCTCGTCAAAGCCATAGTAAGCCTCTTCTTCC
ATATCGGATTCATCATAAATCGGGAATTCTGGAGTCTTGTCTAACAATTCAGCTGTCGCATC
CAAAAGAGTTGCCAAACCTTGCTTGGTCAATCCAGAAATTGGGAAGATGGCTGGAAGTTCC
TCAAACTCGTCGTAGTTTGCTGCCAACTTTTCCTTGAAAGTTTTCAGATTTTCCTGACTTTCA
GGCATATCCATCTTGTTGGCCACGATAATCTGTGGACGTTCCATGAGACGAAGGTTGTAGG
ATTCTAATTCCTTATTGATAGCAAGGTAGTCCTCATAAGGATCACGTCCTTCACTAGCTGAC
ATATCGATGACATGGAGGATAACTCGAGTGCGTTCGATATGACGAAGAAACTGGGTTCCAA
GACCAACCCCTTGACTGGCCCCTTCAATCAAACCTGGAAGGTCTGCTACTGCAAAAGATTC
ACCTGACTGTGTACGAACCATACCCAAATTTGGGACAATCGTCGTAAAGTGGTAAGCACCG
ATTTTGGGCTTAGCTGAAGTGATGACACTAAGCAAAGTTGATTTTCCAACAGATGGGAAAC
CGACCAAGCCAACATCCGCTAAGATTTTCAATTCCAGTTGCAACTCGCGTTCTTGTCCTGG

TTCTCCATTCTCAGAGATTTCAGGTGCAGGATTTTTGGGAGTGGCAAAACGGATATTTCCA
CGACCACCTCGACCGCCATGAGCCACGATAAATTCCTGACCATGTTCAATCAAGTCTGTAA
TGACTTTGCCAGTTTCTGCATCACGTACAGTAGTCCCCTGTGGCACGCGAACACGTAGATC
TTCTGCACCACGTCCGTGCATTCCTTTGGTCATTCCTTTTTCACCGGAATCAGCCTTGAAAT
GACGGTTATAGCGGAAATCCATCAAGGTACGTAAGCCTTCGTCTACCACGAAAACAACGTT
ACCTCCACGTCCACCATCACCACCCCAAGGACCGCCATTAGGGACATATTTTTCACGGCGA
AAGGCAACCATGCCATCGCCACCATTACCAGCCTTGACCTTAATCTTGGCTGTATCTAAAA
ACAT

>HA06_Intergenic_Contig_13

TTATTTCTTTGACTGTGTTTCTTCTTTCTTTGTACGTGCCAATCGAAGAGCGCGTTTAGGATT
GAGACGATTAAATAATTCTTCTAGTTTCTTTTCATTCCATACATCTGCTGCAGAAACAAAATT
GCCATCTGCATCTCGGAATGATATCGGTTTATCTCCCATATCAATTTCCTAGTCTACAAATT
CAAATTCAAATTTACCAATGCGGACCAAATCGCCATCTTTGGCGCCACGCGCACGAAGGG
CTTCGTCAACTCCCATACCACGAAGCTGACGGGCAAACTTCATGACCGATTCGTCACGGTC
AAAGTTGGTCATGTTAAAGAGTTTCATGAGTTTTTCACCAGAAAGTACCCATGTCGCATCAT
CATCACGACTAATCTCAAAGGCTTTTTCTTCCTCGTCAAAGCCATAGTAAGCCTCTTCTTCC
ATATCGGATTCATCATAAATCGGGAATTCTGGAGTCTTGTCTAACAATTCAGCTGTCGCATC
CAAAAGAGTTGCCAAACCTTGCTTGGTCAATCCAGAAATTGGGAAGATGGCTGGAAGTTCC
TCAAACTCGTCGTAGTTTGCTGCCAACTTTTCCTTGAAAGTTTTCAGATTTTCCTGACTTTCA
GGCATATCCATCTTGTTGGCCACGATAATCTGTGGACGTTCCATGAGACGAAGGTTGTAGG
ATTCTAATTCCTTATTGATAGCAAGGTAGTCCTCATAAGGATCACGTCCTTCACTAGCTGAC
ATATCGATGACATGGAGGATAACTCGAGTGCGTTCGATATGACGAAGAAACTGGGTTCCAA
GACCAACCCCTTGACTGGCCCCTTCAATCAAACCTGGAAGGTCTGCTACTGCAAAAGATTC
ACCTGACTGTGTACGAACCATACCCAAATTTGGGACAATCGTCGTAAAGTGGTAAGCACCG
ATTTTGGGCTTAGCTGAAGTGATGACACTAAGCAAAGTTGATTTTCCAACAGATGGGAAAC
CGACCAAGCCAACATCCGCTAAGATTTTCAATTCCAGTTGCAACTCGCGTTCTTGTCCTGG
TTCTCCATTCTCAGAGATTTCAGGTGCAGGATTTTTGGGAGTGGCAAAACGGATATTTCCA
CGACCACCTCGACCGCCATGAGCCACGATAAATTCCTGACCATGTTCAATCAAGTCTGTAA
TGACTTTGCCAGTTTCTGCATCACGTACAGTAGTCCCCTGTGGCACGCGAACACGTAGATC
TTCTGCACCACGTCCGTGCATTCCTTTGGTCATTCCTTTTTCACCGGAATCAGCCTTGAAAT
GACGGTTATAGCGGAAATCCATCAAGGTACGTAAGCCTTCGTCTACCACGAAAACAACGTT
ACCTCCACGTCCACCATCACCACCCCAAGGACCGCCATTAGGGACATATTTTTCACGGCGA
AAGGCAACCATGCCATCGCCACCATTACCAGCCTTGACCTTAATCTTGGCTGTATCTAAAA
ACAT

>1647_Intergenic_Contig_17

TTATTTCAATTTATCTGCTTCATAGGTGTGAACAAGCTCAAGACCTGCAAAGTTCTGCTGGC
GGAGGGCTTCATAAACAATCATGCATACGGTATTCGAAACATTGAGGCTGCGAACGTGTTC
ATCGTTCATAGGAATACGGAGAGCCTTCTCAGGATGGTCTCGCATAAAGTCCTCGGGCAA
GCCCTTGTCTTCACGTCCAAAGAGAAAATAATGGTCTTCGTCAGTCGATAAATCCACCTCA
GAATACACTTTCTCAGCAAATTTCGAAATCAGATAGACTTGTCCTTTCATCTGAGACATGAA
ATCTTCCAAGCTGTCATAAAAATAAATCTCAAGCTTATCCCAGTAATCCAAACCAGCCCGCT
TCATCTTTCGGTCGTCAATGGGAAAGCCCATTGGTTTAATGATGTGGAGGGGAGAATTGGT
CGCAGCGCAAGTACGCGCAATGTTACCTGTATTTTGTGGAATCTGAGGCTCGAATAATACA
ATGTGATTTGTCATGACGGGTTTCCTTTTATCATTGCAAAAAAATAGCCACACTGCCCGGAG
TCAAGCTCAGCAAACAGCGTGGTTAAGGCGTCGTTAACTTACCTCACAACAGGTTTGAAGT
AAATCAGCGAAACTACTTCCTTAGTATAACACTTTCAGAATCATTGTCAATAGAAACGACTT
GATTTTTTTACTTTTTTTGATGATATTTTCATGTTTGGTCATTCCTTTCTGAAAAGGTATGGTA

AAAGAGCCAAATCACTTCAAATCAGAGACATTGTTATAGAAGTTAAGCAAATGTATCTTCAA
AACTTGAAAAAAGCCTTTCTTCCCCTAAAAATCGCCTTAGAAAGTGCATTTTTTGCTAAAAAA
GGGTATGATAGTTACATCATGAAAAAGTAG

>HA06_Intergenic_Contig_17

TTATTTCAATTTATCTGCTTCATAGGTGTGAACAAGCTCAAGACCTGCAAAGTTCTGCTGGC
GGAGGGCTTCATAAACAATCATGCATACGGTATTCGAAACATTGAGGCTGCGAACGTGTTC
ATCGTTCATAGGAATACGGAGAGCCTTCTCAGGATGGTCTCGCATAAAGTCCTCGGGCAA
GCCCTTGTCTTCACGTCCAAAGAGAAAATAATGGTCTTCGTCAGTCGATAAATCCACCTCA
GAATACACTTTCTCAGCAAATTTCGAAATCAGATAGACTTGTCCTTTCATCTGAGACATGAA
ATCTTCCAAGCTGTCATAAAAATAAATCTCAAGCTTATCCCAGTAATCCAAACCAGCCCGCT
TCATCTTTCGGTCGTCAATGGGAAAGCCCATTGGTTTAATGATGTGGAGGGGAGAATTGGT
CGCAGCGCAAGTACGCGCAATGTTACCTGTATTTTGTGGAATCTGAGGCTCGAATAATACA
ATGTGATTTGTCATGACGGGTTTCCTTTTATCATTGCAAAAAAATAGCCACACTGCCCGGCG
TCAAGCTCAGCAAACAGCGTGGTTAAGGCGTCGTTAACTTACCTCACAACAGGTTTGAAGT
AAATCAGCGAAACTACTTCCTTAGTATAACACTTTCAGAATCATTGTCAATAGAAACGACTT
GATTTTTTTACTTTTTTTGATGATATTTTCATGTTTGGTCATTCCTTTCTGAAAAGGTATGGTA
AAAGAGCCAAATCACTTCAAATCAGAGACATTGTTATAGAAGTTAAGCAAATGTATCTTCAA
AACTTGAAAAAAGCCTTTCTTCCCCTAAAAATCGCCTTAGAAAGTGCATTTTTTGCTAAAAAA
GGGTATGATAGTTACATCATGAAAAAGTAG

***S. oralis* 1648/RA08**

>1648_cdsA

MTKDLQKRTLFAVLALAIFLPVLFAGGLLLQIGIGLLAMLGVHELLHMKGLKTMTIEGALTLFATFA
LTVPLENYLTFLPVDGNVVAYSVLITIMLGTTVFSKNYTIEDAAFPIAVSFYVGFGFNALLDARVA
GFDKVLLALFIVWATDSAAYLTGMNFGKHKLAPRVSPNKSIEGFVGGILGAVLITVIFMLVDSTVA
LPYGIYRMSLFAAFFSVAGQFGDLIESAAKRHFGVKDSGKFIPGHGGVLDRFDSMLIVFPMMHL
FGLF

>HA08_cdsA

MTKDLQKRTLFAVLALAIFLPVLFAGGLLLQIGIGLLAMLGVHELLHMKGLKTMTIEGALTLFATFA
LTVPLENYLTFLPVDGNVVAYSVLITIMLGTTVFSKNYTIEDAAFPIAVSFYVGFGFNALLDARVA
GFDKVLLALFIVWATDSAAYLTGMNFGKHKLAPRVSPNKSIEGFVGGILGAVLITVIFMLVDSTVA
LPYGIYRMSLFAAFFSVAGQFGDLIESAAKRHFGVKDSGKFIPGHGGVLNRFDSMLIVFPMMHL
FGLF

>1648_ppGpp_synthetase

MPKEVNLTGDQVVALTKEYLTKEDVAFVQKALIYAVDCHSGQFRKSGEPYIIHPIQVAGILAKLKL
DAVTVACGFLHDVVEDTDATLDDLEREFGHDVRIIVDGVTKLGKVEYKSLEEQLAENHRKMLM
AMSEDIRVILVKLSDRLHNMRTLKHLRKDKQERISRETMEIYAPLAHRLGISSVKWELEDLSFRY
LNPTEFYKITHMMKEKRREREALVDEVVTKLEDYATERNLKGKIYGRPKHIYSIYRKMQDKKKR
FEEIYDLIAIRCILDTQSDVYAMLGYVHELWKPMPGRFKDYIANRKANGYQSIHTTVYGPKGPIE
FQIRTKEMHEVAEYGVAAHWAYKKGIKGQVNSKESAIGMNWIKEMMELQDQADDAKEFVDSV
KENYLAEEIYVFTPDGAVRSLPKDSGPIDFAYEIHTKVGEKATGAKVNGRMVPLTTKLKTGDQV

EIITNPNSFGPSRDWLNMVKTSKARNKIRQFFKNQDKELSVNKGREMLMAQFQENGYVANKF
MDKRHMDEVLQKTSYKTEEALYAAIGFGEIGAITVFNRLTEKERREEERAKAKAEAEELVKGGE
VKVENKETLKVKHEGGVVIEGASGLLVRIAKCCNPVPGDDIVGYITKGRGVAIHRVDCMNLRAQ
ENYEQRLLDVEWEDQFSSKEYTAHIDIYGLNRTGLLNDVLQVLSNTTKNISTVNAQPTKDMKFA
NIHVSFGISNLSTLTTVVDKIKSVPEVYAVKRTNG

>HA08_ppGpp_synthetase

MPKEVNLTGDQVVALTKEYLTKEDVAFVQKALIYAVDCHSGQFRKSGEPYIIHPIQVAGILAKLKL
DAVTVACGFLHDVVEDTDATLDDLEREFGHDVRIIVDGVTKLGKVEYKSLEEQLAENHRKMLM
AMSEDIRVILVKLSDRLHNMRTLKHLRKDKQERISRETMEIYAPLAHRLGISSVKWELEDLSFRY
LNPTEFYKITHMMKEKRREREALVDEVVTKLEDYATERNLKGKIYGRPKHIYSIYRKMQDKKKR
FEEIYDLIAIRCILDTQSDVYAMLGYVHELWKPMPGRFKDYIANRKANGYQSIHTTVYGPKGPIE
FQIRTKEMHEVAEYGVAAHWAYKKGIKGQVNSKESAIGMNWIKEMMELQDQADDAKEFVDSV
KENYLAEEIYVFTPDGAVRSLPKDSGPIDFAYEIHTKVGEKATGAKVNGRMVPLTTKLKTGDQV
EIITNPNSFGPSRDWLNMVKTSKARNKIRQFFKNQDKELSVNKGREMLMAQFQENGYVANKF
MDKRHMDEVLQKTSYKTEEALYAAIGFGEIGAITVFNRLTEKERREEERAKAKAEAEELVKGGE
VKVENKETLKVKHEGGVVIEGASGLLVRIAKCCNPVPGDDIVGYITKGRGVAIHRVDCMNLRAQ
ENYEQRLLDVEWEDQFSSKEYTAHIDIYGLNRTGLLNDVLQVLSNTTKNISTGNAQPTKDMKFA
NIHVSFGISNLSTLTTVVDKIKSVPEVYAVKRTNG

>1648_Ribosomal_SSU_MTase

VTKVETARSLALAVLEDVLMNQAYSNIALNKHLKGSQLSVADKGLVTEIVYGTVARKLTLEWYLS
HFIEDRDKLDNWLYVLLLLSAYQLRYLDKIPNHAVVNEAVELAKARKKGSEKLVNAVLRRILREG
WPDIDSIKRKNKRDSIAYSLPVWLVSKLKEEYGEERAQAIFESLLVRNKASIRVTDLSRKEEIKAL
LEATDSPLVATGLVKEHGHFAGHDLFADGAITIQDESSQLVAPTLDLQGDERVLDACAAPGGKT
AHIASYLTTGQVTALDLYDHKLDLIQENAERLGVADRVQTQKLDARKVHEFFGRDSFDKILVDA
PCSGIGLLRRKPDIKYNKETADFTSLQEIQLEILGSVCQTLRKGGIITYSTCTIVSEENFQVVEAFL
ESHPEFEQVKLEHECKDILKDGCILITPELYGSDGFFISQFRKISE

>HA08_Ribosomal_SSU_MTase

VTKVETARSLALAVLEDVLMNQAYSNIALNKHLKGSQLSVADKGLVTEIVYGTVARKLTLEWYLS
HFIEDRDKLDNWLYVLLLLSAYQLRYLDKIPNHAVVNEAVELAKARKKGSEKLVNAVLRRILREG
WPDIDSIKRKNKRDSIAYSLPFWLVSKLKEEYGEERAQAIFESLLVRNKASIRVTDLSRKEEIKAL
LEATDSPLVATGLVKEHGHFAGHDLFADGAITIQDESSQLVAPTLDLQGDERVLDACAAPGGKT
AHIASYLTTGQVTALDLYDHKLDLIQENAERLGVADRVQTQKLDARKVHEFFGRDSFDKILVDA
PCSGIGLLRRKPDIKYNKETADFTSLQEIQLEILGSVCQTLRKGGIITYSTCTIVSEENFQVVEAFL
ESHPEFEQVKLEHECKDILKDGCILITPELYGSDGFFISQFRKISE

>1648_Helicase_PriA

MAIAKIIVNVPLMQTDQPYSYKVPEEFIDMLEVGMRVHVPFGKGNRLIQGIVLGLESQSDEEVAD
QNLKEIAEVLDFSPVLTQEQLWLAEELRKSVFSYKISILKAMLPGFLNSSYDKILYPLEGLSQADR
ERLFGSEDSLAFSSLDLAKQAEMMRLTRKGLLRLEYQAVDQKKVKTQSWYEVDATQLEKIEISA
RAKKKAELRDYLLSHPKSAPLASLLESYSREQVNFFVEQGAVSIVQKEVQRSAAYFEGIEASQP
LELNPEQRKARDAVVSAIGSQQPPFLLQGITGSGKTEVYLQIIQGALDKGKTAILLVPEISLTPQM
TERFVARFGEKVAILHSGLSNGEKYDEWRKVERGDAQVVVGARSAIFAPLKNLGVMIIDEEHEA
SYKQDSNPRYHAREVAILRAQYNQAALVLGSATPSLESRARAGKGVYQHLRLTQRANPLATIP
EVQVIDFRDYIGQNETSNFTPPLLEAIQDRLDKKEQVVLMLNRRGYSSFVMCRECGTVDSCPN

CDISLTLHMDTKTMNCHYCGFSKGIPHVCPNCQSRSIRYYGTGTQKAYDELVELFPQARILRMD
VDTTRKKGSHQALLEQFGRGEADILLGTQMIAKGLDFPNVTLVGVLNADTALNLPDFRSSERTF
QLLTQVAGRAGRAEKTGQVLIQSYNPEHYAIRFAKDQDYEGFYAYEMSIRRQLGYPPYYFTIGI
TLSHKKEEEVVKRAYEVMGILRSGLSETSKILGPTPKPIARTHNLYHYQILIKYRLEDELGPTLNR
VLALTQERENSELRLSIDHEPQQFL

>HA08_Helicase_PriA

MAIAKIIVNVPLMQTDQPYSYKVPEAFIDMLEVGMRVHVPFGKGNRLIQGIVLGLESQSDEEVAD
QNLKEIAEVLDFSPVLTQEQLWLAEELRKSVFSYKISILKAMLPGFLNSSYDKILYPLEGLSQADR
ERLFGSEDSLAFSSLDLAKQAEMMRLTRKGLLRLEYQAVDQKKVKTQSWYEVDATQLEKIEISA
RAKKKAELRDYLLSHPKSAPLASLLESYSREQVNFFVEQGAVSIVQKEVQRSAAYFEGIEASQP
LELNPEQRKARDAVVSAIGSQQPPFLLQGITGSGKTEVYLQIIQGALDKGKTAILLVPEISLTPQM
TERFVARFGEKVAILHSGLSNGEKYDEWRKVERGDAQVVVGARSAIFAPLKNLGVMIIDEEHEA
SYKQDSNPRYHAREVAILRAQYNQAALVLGSATPSLESRARAGKGVYQHLRLTQRANPLATIP
EVQVIDFRDYIGQNETSNFTPPLLEAIQDRLDKKEQVVLMLNRRGYSSFVMCRECGTVDSCPN
CDISLTLHMDTKTMNCHYCGFSKGIPHVCPNCQSRSIRYYGTGTQKAYDELVELFPQARILRMD
VDTTRKKGSHQALLEQFGRGEADILLGTQMIAKGLDFPNVTLVGVLNADTALNLPDFRSSERTF
QLLTQVAGRAGRAEKTGQVLIQSYNPEHYAIRFAKDQDYEGFYAYEMSIRRQLGYPPYYFTIGI
TLSHKKEEEVVKRAYEVMGILRSGLSETSKILGPTPKPIARTHNLYHYQILIKYRLEDELGPTLNR
VLALTQERENSELRLSIDHEPQQFL
